# Growth optimization and dual cellulolytic degradation pathways in *Serpula lacrymans*

**DOI:** 10.1186/s13568-026-02009-5

**Published:** 2026-02-08

**Authors:** Katsiaryna Kakhanouskaya, Aliaksandr Kakhanouski

**Affiliations:** Scientific Research Group of Agricultural Company “Staletava”, Mahilioŭ, Belarus

## Abstract

*Serpula lacrymans* is the most destructive brown-rot fungus affecting timber in temperate regions, causing severe economic losses in construction and cultural heritage. Conventional chemical treatments are largely ineffective, as they act only on wood surfaces and fail to eradicate fungal growth within deeper layers. Previous studies have described the cellulolytic activity of *S. lacrymans* as weak and strictly substrate-inducible, despite genomic evidence for GH12-type endoglucanases. Moreover, cultivation in traditional malt- or CMC-based media often yields insufficient biomass, limiting reproducibility of biochemical assays and obscuring the true enzymatic spectrum. Here, we report that cultivation in Murashige–Skoog salt medium supplemented with sucrose markedly improves biomass yield and metabolite accumulation, enabling reliable physiological and enzymatic analyses. Our findings reveal basal endoglucanase activity and demonstrate that *S. lacrymans* employs both hydrolytic and Fenton-like oxidative mechanisms in cellulose degradation. These insights advance the understanding of its decay system and provide a foundation for developing biological control strategies against this highly destructive fungus.

## Introduction

*Serpula lacrymans* is one of the most destructive fungi affecting timber in buildings. Once established, it causes severe structural damage by rapidly degrading cellulose and hemicellulose, leaving behind brittle, weakened wood. The fungus is notoriously difficult to eradicate: conventional chemical treatments penetrate only the surface layers of timber and fail to reach the deeper mycelial networks, which continue to grow and spread. As a result, infestations often recur even after chemical intervention. For this reason, biological approaches to control are considered a promising alternative. Developing such strategies requires a detailed understanding of the biochemical and physiological characteristics of *S. lacrymans*. At present, there are significant gaps in knowledge regarding its enzyme systems, oxidative mechanisms, and nutritional requirements.

In laboratory cultivation of basidiomycetous fungi, traditional nutrient agar formulations such as malt extract agar or Czapek’s synthetic medium are widely used (Jennings 1982; Maurice 2011). However, these media were primarily designed for molds and yeast-like saprotrophs and do not fully meet the mineral requirements of wood-decaying basidiomycetes such as *S. lacrymans*. Low biomass yields in malt-based media are often linked to deficiencies in macro- and micronutrients. For this reason, it was necessary to search for an optimized growth medium. Also cultivating the fungus under balanced nutritional conditions is crucial for biochemical investigations because it ensures sufficient biomass for experimental analyses, it stimulates enzyme expression and secretion, allowing the spectrum and activity of cellulolytic enzymes to be revealed and increases the reliability of results, since nutrient limitations may obscure or distort the actual degradation mechanisms. Thus, optimization of the growth medium not only improves the cultivation of *S. lacrymans* but also establishes the necessary foundation for deeper biochemical and physiological studies.

At present, there is no direct evidence that *S. lacrymans* exhibits clearly constitutive endoglucanase activity. Most studies have described its cellulolytic activity as weak and strictly substrate-inducible, for example in the presence of carboxymethyl cellulose (CMC) (Eastwood 2011; Zhu 2020). Nevertheless, the genome of *S. lacrymans* contains genes belonging to the GH12 family, and several authors have noted that certain hydrolytic enzymes in brown-rot fungi may display a low basal level of expression that could be interpreted as constitutive (Eastwood 2011; Elisashvili 2002; Zhu 2020). This remains a hypothesis that requires further experimental validation (Goodell 2017; Umezawa 2020; Zhu 2020). Understanding the inducibility of enzyme systems is therefore essential, as it provides insight into ecological adaptability and helps to identify strategic conditions under which such systems may be most effectively targeted or disrupted.

The objectives of this study were, first, to demonstrate that a medium traditionally used for the cultivation of plant cells can also be successfully applied to the growth of wood-decaying saprotrophic fungi, using *S. lacrimans* as a model organism. Second, to provide evidence of endoglucanase activity in the culture liquid of this fungus, thereby confirming not only its enzymatic capacity for cellulose degradation but also its broad adaptive strategy to utilize diverse substrates under varying nutritional conditions.

## Materials and methods

### Strain origin and deposition

*S. lacrymans* used in this study obtained from the museum collection of the Institute of Experimental Botany, where it had previously been identified by experienced mycologists (MSU–F19463 (Herbarium of V.F. Kuprevich Institute of Experimental Botany, NASB). The Institute of Experimental Botany deposited the specimen in the reference collection of the Institute of Microbiology (Belarusian Collection of Non-pathogenic Microorganisms as *S. lacrymans* BIM F-835). In this work, the strain is designated as *S. lacrymans* S35.

### Culture media and incubation conditions

Three artificial media were used to evaluate fungal growth and enzymatic activity:

- Malt agar: 50 g/L malt extract, 20 g/L agar, pH 6.0
- Murashige and Skoog (MS) medium: salt solution + 35 g/L sucrose, 20 g/L agar, pH 6.0
- MS supplemented with sodium–carboxymethyl cellulose (Na–CMC): same MS salt base + 35 g/L Na–CMC, 20 g/L agar, pH 6.0

For submerged cultivation, agar was omitted. Colony diameter was measured over 9–12 days to assess hyphal growth dynamics. All experimental cultures of the *S. lacrymans* S35 strain were maintained under a constant incubation temperature of 20 °C, which corresponds to the optimal growth conditions for xylotrophic basidiomycetes associated with brown rot. This temperature was selected in accordance with the ecological profile of the species, aiming to ensure maximal uniformity in the biological characteristics of the culture filtrates intended for analysis of both enzymatic and non-enzymatic activity.

To assess biological variability, different fragments of mycelium derived from a single clonal isolate were used, each exhibiting distinct biochemical activity. Each fragment was cultured independently, allowing them to be considered as separate biological replicates.

### Endoglucanase activity assay

To assess cellulolytic activity, changes in the viscosity of CMC solution (Sharrock 1988) were measured following the addition of culture fluid obtained from *S. lacrymans* under different growth conditions. Control experiments included comparison with buffer-only samples, ensuring that the observed changes were attributable to enzymatic effects. Because viscosity is strongly influenced by temperature, pH, and the degree of dilution with culture fluid, all these parameters were strictly controlled during the experiments to guarantee reproducibility and to ensure that the measured changes reflected enzymatic activity rather than external factors.

Enzymatic activity of the culture liquid was quantified via dynamic viscosity change of 2.5% Na-CMC solution (pH 6.8, 20 °C) at 1 and 2 h after reaction onset. Mycelia were incubated in 50 mL liquid medium (same composition as solid media, minus agar). After cultivation, 1 mL of culture liquid was added to 50 mL of CMC solution prepared with Murashige–Skoog salts. Viscosity (η) was calculated using the formula:

$$ \eta = \left( {2/9} \right) \times r^{2} \times g \times (\rho_{0} - \rho )/V $$

r: radius of lead ball = 0.001 m.

g: gravity acceleration = 9.81 m/s^2^

*ρ*_0_: density of lead ball = 11,350 kg/m^3^

*ρ*: density of CMC solution (kg/m^3^).

V: velocity of fall (h = 0.0097 m).

Reynolds number (Re) was calculated to exclude turbulent values:

$$ {\mathrm{Re}} = \rho \times V \times d/\eta $$

d: ball diameter (m).

Measurements with Re ≥ 1 were excluded from mean calculations. Also we employed filtrates lacking enzymatic activity (obtained from cultures grown on non-inducing substrates) as negative controls. This was essential because the viscometric method requires a very careful approach to distinguish true cellulolytic activity from possible non-specific protein effects on CMC solution viscosity. It should be noted that in the experiment we considered not the absolute value of dynamic viscosity, but only its change over time, since variations in the chemical composition of the culture liquid could also influence the magnitude of the absolute dynamic viscosity of the CMC solution. The results were normalized to 1 mg of total protein in the culture fluid.

### Protein quantification

Protein concentration in the culture liquid was measured using the Biuret method, calibrated with BSA standards. We did not employ the Lowry (Folin–Ciocalteu) method because our culture medium (wort) contained high amounts of mono‑ and disaccharides. These reducing sugars are known to interfere with the Folin reagent, resulting in overestimation of protein concentration. Direct UV absorbance at 280 nm was not used, since the Murashige–Skoog medium contains various salts (e.g., nitrates, phosphates, sulfates) that absorb in the UV range and interfere with accurate protein quantification. Therefore, we chose the Biuret assay. Although less sensitive than Lowry or Bradford, the Biuret method is more robust in complex media, being less affected by reducing sugars abundant in wort and by salts present in Murashige–Skoog medium. The Biuret reaction provides a direct measure of total protein content through the formation of a copper–protein complex, and thus offered a reliable approach under our experimental conditions.

### Statistical analysis

All experiments were performed with 3–6 independent biological replicates. Normality was assessed using the Kolmogorov–Smirnov test; variance homogeneity was tested via Levene’s test. Parametric tests (Student’s t-test) were applied to datasets with normal distribution and equal variances. Non-parametric tests (Mann–Whitney for two groups, Kruskal–Wallis for three groups) were used where assumptions were violated. Significance threshold (*α*) was 0.05. Analysis was performed in Excel 2019 and Statistica 10.

## Results

To evaluate mycelial growth efficiency, three types of solidified media were tested: MS (Murashige–Skoog solution with 3.5% sucrose), wort agar (based on 3.5°B barley malt extract), and MS–CMC (Murashige–Skoog solution with 3.5% carboxymethyl cellulose as the sole carbon source).

**Fig. 1:**
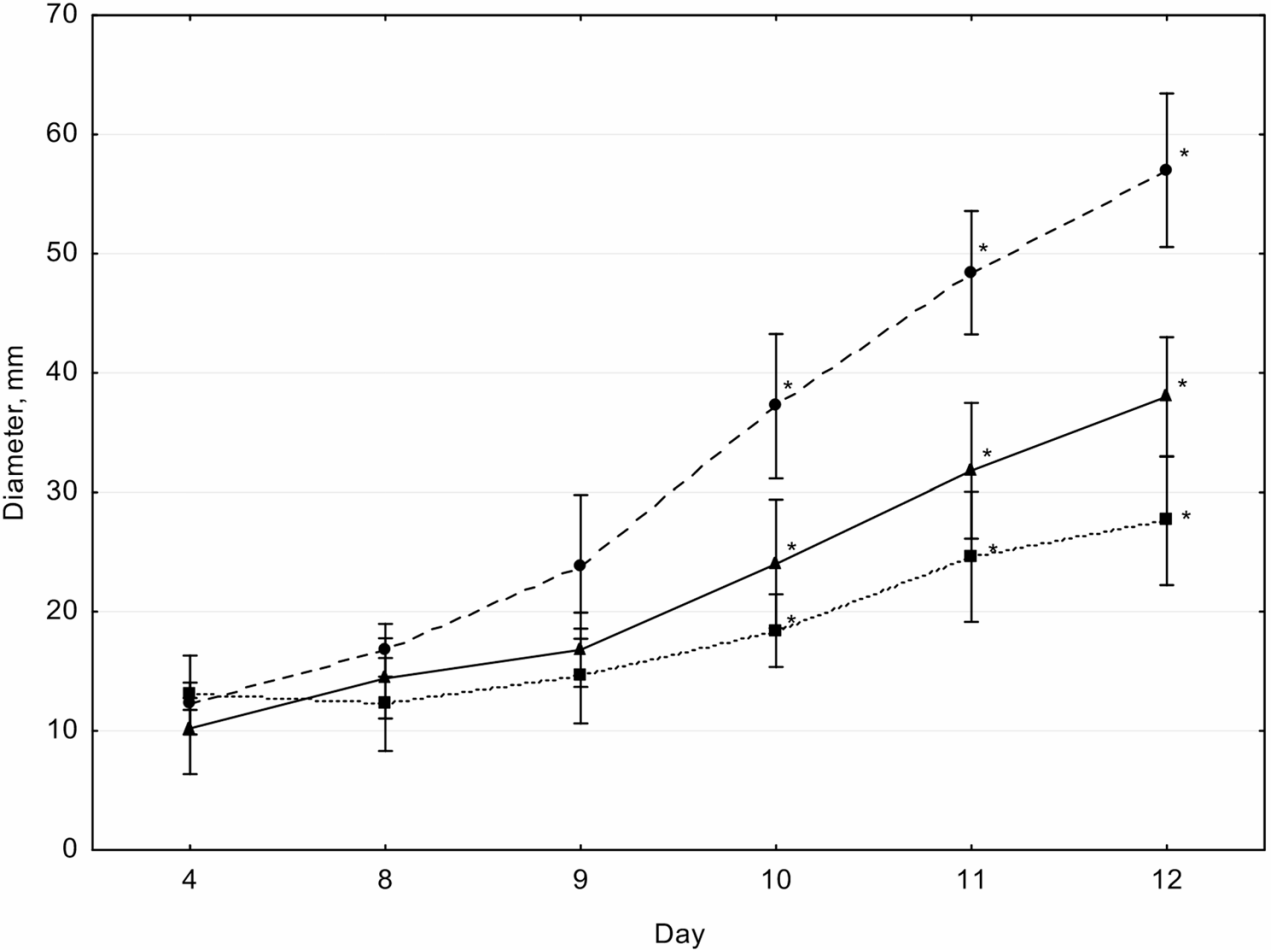
Diameter of *S. lacrymans* S35 colonies on different solidified media during 12 days of cultivation. Data represent n = 5 biological replicates. Values are expressed as mean ± SD. Statistically significant differences marked by asterisks, Kruskal–Wallis test: H_*exp*_ = 10.1; H_*crit*_ = 5.66; *α* = 0.05). Solid line – wort agar (3.5°B, 2% agar); dashed line – MS medium (Murashige–Skoog salts with 3.5% sucrose, 2% agar); dotted line – MS–CMC medium (Murashige–Skoog salts with 2.5% CMC, 2% agar)

Colony growth analysis (Fig. 1) revealed the largest colony diameter on MS medium, while the lowest growth was observed on MS–CMC. From day 10 onward, the differences in colony diameters across media were statistically significant (Kruskal–Wallis test: H_exp_ = 10.1; H_crit_ = 5.66; α = 0.05).

**Fig. 2:**
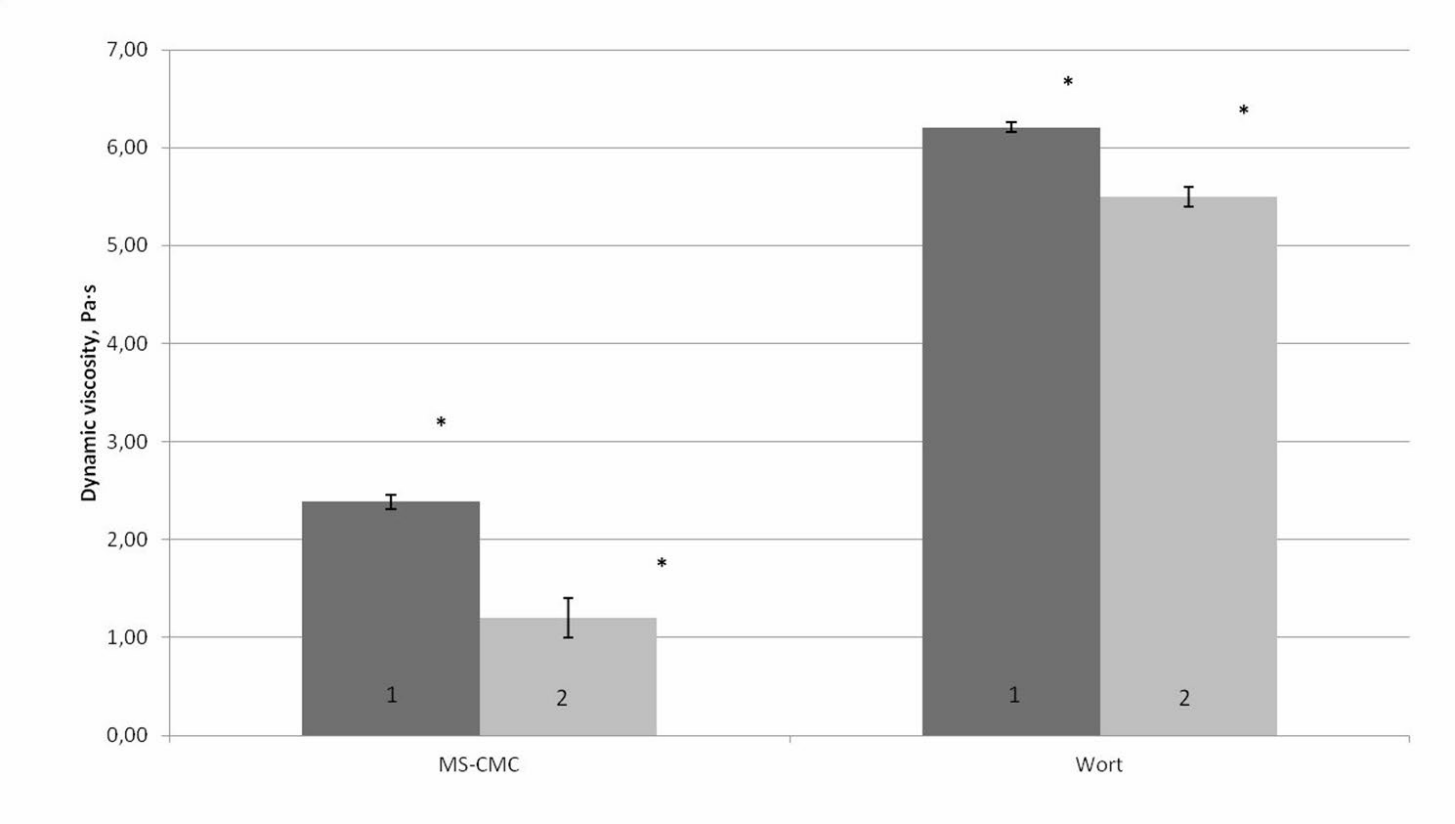
Changes in dynamic viscosity of CMC solution after incubation with *S. lacrymans S35* culture filtrates. Red bars represent 1 h exposure; pink bars represent 2 h exposure. Data represent n = 6 biological replicates. Values are expressed as mean ± SD. Statistical significance was determined using the Mann–Whitney test (*p* < 0.00001)

To assess the presence of endoglucanase activity, mycelia were cultivated in liquid media: 3.5°B wort broth and MS–CMC (Murashige–Skoog solution with 2.5% CMC as the sole carbon source). Culture filtrates were sampled after the active growth phase, ensuring sufficient protein concentration and stable physicochemical parameters suitable for enzymatic assays. Viscosity tests of 2.5% CMC solutions (Fig. 2) showed statistically significant reduction after incubation with culture filtrates from both media types, confirming the presence of endoglucanase activity. Filtrates derived from CMC-based cultures exhibited significantly higher activity (n = 6 biological replicates; Mann–Whitney test, *p* < 0.00001), supporting the interpretation that cellulolytic activity in *S. lacrymans* is substrate-inducible.

**Fig. 3:**
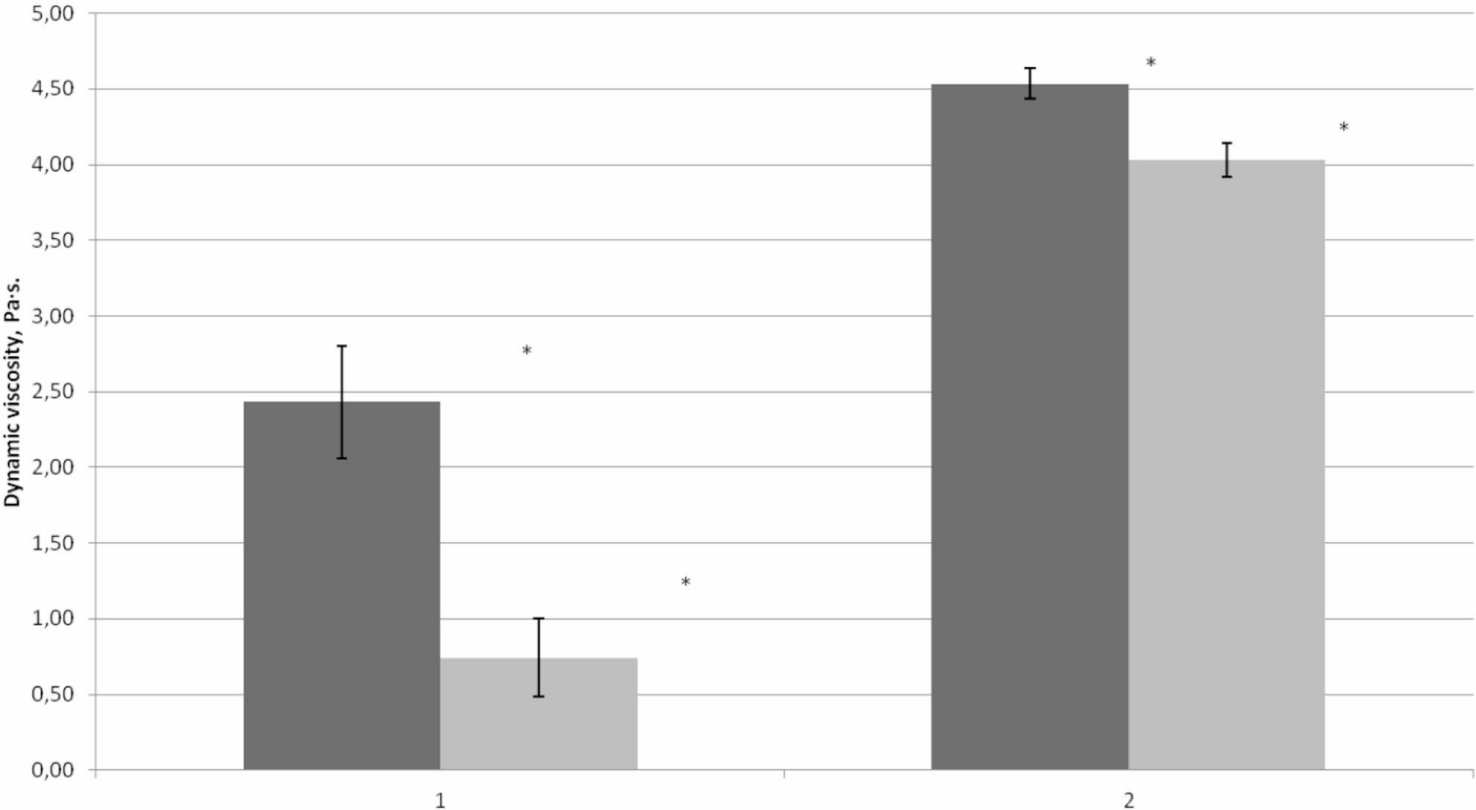
CMC solution dynamic viscosity after addition of native and heat-treated *S. lacrymans* S35 filtrates (1) – native, (2) – heat-treated. Dark grey (left) column – initial viscosity; grey (right) column – after 30 min. Mean ± SD shown; statistically significant differences indicated (Mann–Whitney test; n = 5; *p* < 0.00033 for native, *p* < 0.00672 for heat-treated)

To quantify degradation mechanisms, culture filtrates—native and heat-denatured—were tested on 2.5% CMC solution (Fig. 3) with cultural supernatants derived from *S. lacrymans* grown in media containing simple sugars. Viscosity reduction was more pronounced for native filtrates compared to heat-treated ones (Mann–Whitney test, *p* < 0.00033, for native, *p* < 0.00672 for heat-treated), indicating a partial loss of CMC-degrading (endoglucanase) activity in the heat-treated samples. Independent biological replicates (n = 5) ensured robustness and reproducibility.

## Discussion

### Mycelial growth of* S. lacrymans* S35 across different nutrient media

As noted previously, laboratory cultivation of basidiomycetous fungi has traditionally relied on nutrient agar media such as malt extract agar and Czapek’s synthetic medium (Jennings 1982; Maurice 2011), which are primarily optimized for the growth of molds and yeast-like saprotrophs. However, basidiomycetes, including the saprotrophic fungus *S. lacrymans*, exhibit more specific requirements for mineral components due to their natural ecological niche—wood. According to literature, low biomass yields in malt-based media are attributed to deficits in macro- and micronutrients (Aleksandrova 1998).

To address this limitation, we sought to identify a synthetic medium optimal for cultivating *S. lacrymans* S35. Being a saprotrophic fungus that colonizes dead plant tissue, *S. lacrymans* utilizes not only cellulose but also a wide array of mineral nutrients present in lignocellulosic substrates. Consequently, nutrients absorbed by plant cells from their growth medium are subsequently incorporated into fungal mycelia through the trophic chain. This rationale suggests that the Murashige–Skoog medium, originally formulated for plant cell culture and containing a comprehensive profile of macro- and micronutrients, may serve as a more appropriate synthetic substrate for *S. lacrymans* cultivation.

Our experimental results support this hypothesis: the Murashige–Skoog medium supplemented with sucrose significantly enhanced mycelial growth under laboratory conditions. This medium potentially ensures improved biomass yield and facilitates accumulation of target metabolites for downstream biochemical assays.

These findings indicate that the use of malt extract medium for cultivating basidiomycetous fungi, including *S. lacrymans*, may be limited due to insufficient mineral content. Although malt-based media provide essential carbon sources such as maltose, glycerol, and dextrin, they are deficient in nitrogen and critical minerals—limiting their efficacy for fungi with pronounced mineral requirements (Aleksandrova 1998). In contrast, the salt composition of the Murashige–Skoog medium offers a balanced nutrient profile conducive to the growth of xylotrophic fungi.

### Viscometric assessment of endoglucanase activity

The viscometric method is a reliable and straightforward tool for determining cellulase activity, particularly that of endoglucanases (Sharrock 1988). It enables rapid assessment of enzymatic activity through the reduction in viscosity of carboxymethylcellulose solutions. This approach is especially sensitive to the early stages of hydrolysis, since even minor reductions in the degree of polymerization lead to a noticeable decrease in solution viscosity (Lee 2007).

In the case *of S. lacrimans*, exoglucanase activity is known to be very weak (Hori 2013), which makes methods based on the detection of reducing sugars unsuitable for revealing the enzymatic pathway of cellulose degradation. Although expression of endoglucanase genes has been documented in this fungus, corresponding enzymatic activity in the culture liquid has not been detected earlier. These enzymes cleave CMC without producing monosaccharides, yielding instead polymers of lower molecular weight while the overall amount of CMC in solution remains unchanged. Consequently, detection of reducing sugars is complicated, and reliable data can only be obtained by measuring the rheological properties of the polymer solution.

Previous studies have demonstrated that changes in solution viscosity and the accumulation of reducing sugars generally provide comparable results (Sharrock 1988). However, in our case, the absence of monosaccharide formation renders the colorimetric approach unsuitable. To avoid methodological errors and ensure reproducibility and reliability of the results, we carefully selected experimental conditions and employed appropriate controls. The construction of a calibration curve to convert viscosity reduction into standard units of enzymatic activity may lead to systematic errors. This limitation arises from the absence of a direct reaction product, the dependence of results on the concentration and structural properties of CMC, the potential influence of non-specific components present in culture filtrates, and differences between the standard enzyme and the investigated sample. In such assays, activity can be expressed either as the percentage decrease in viscosity relative to the control or as the rate of viscosity loss (Pa·s/min).

Therefore, the viscometric method should be regarded as a reliable tool for assessing relative changes in activity, but not for absolute calibration into standard enzymatic units. However, the viscometric approach is more prone to variations (e.g., dependence on shear rate, temperature, substrate concentration), which limits its accuracy but makes it suitable for comparative analysis and screening (Lee 2007).

### Inducible and constitutive cellulolytic activity and CMF pathway involvement in* S. lacrimans*

Brown rot fungi, including *S. lacrymans*, demonstrate high cellulose-degrading capacity (Nurika 2020) despite possessing a relatively limited set of genes associated with enzymatic polysaccharide hydrolysis. Compared to white rot fungi*, S. lacrymans* genomes exhibit reduced representation of cellulolytic genes such as GH74 (xyloglucanase), GH10 (endoxylanase), GH79 (β-glucuronidase), CE1 (acetylxylan esterase), CE15 (glucuronoyl methylesterase), as well as the absence of cellulose-binding modules CBM1 (Hori 2013; Floudas 2012).

Transcriptomic and proteomic studies (Hori 2013) show that *S. lacrymans* does not express cellobiose dehydrogenase or lytic polysaccharide monooxygenases during growth on wood, although it retains a single copy of the GH6 cellobiohydrolase gene. Nevertheless, cellulolytic activity is carried out via alternative enzymes. In the closely related *S. incrassata*, three extracellular endoglucanases have been identified, suggesting stage-specific differential expression during lignocellulose decomposition (Kleman-Leyer and Kirk 1994).

Cellulose depolymerization in *S. lacrymans* is mediated through two complementary mechanisms: enzymatic and non-enzymatic (Elisashvili 2002; Floudas 2012; Ritschkoff 1990; Suzuki 2006; Zhu 2020). The latter involves a chelator-mediated Fenton pathway, whereby the fungus produces hydrogen peroxide, iron-reducing compounds, and oxalic acid. These contribute to the formation of Fe(III)-oxalate complexes, which under acidic conditions are reduced to Fe(II), subsequently generating hydroxyl radicals that trigger non-enzymatic cleavage of the lignocellulosic matrix. This CMF process accounts for approximately 80–90% of total cellulolytic activity. The remaining 10–20% is attributed to enzymatic activity via GH6/7 glycoside hydrolases and acidic endoglucanases.

In basidiomycetes, cellulase expression may be either constitutive or inducible, with the presence of cellulose substrate acting as a stimulatory trigger (Martinez 2009). Our results confirm that *S. lacrymans* S35 displays inducible activity, evidenced by a significantly greater reduction in CMC solution viscosity when grown on cellulose-based medium versus maltose. This indicates that *S. lacrimans* activates specific mechanisms in response to complex substrates. Previous studies (Nurika 2020) have shown that this fungus possesses genes IR1 and IR2, which are homologous to cellobiose dehydrogenase (CDH) and are capable of reducing Fe⁺ to Fe^2^⁺. As a consequence, a non-enzymatic pathway of cellulose degradation can be initiated through the Fenton system (Fe^2^⁺ + H_2_O_2_ → ·OH), in which hydroxyl radicals cause oxidative damage and depolymerization of cellulose.

The expression of iron reductases in *S. lacrymans* is expected to be suppressed during growth on malt, since their synthesis is inhibited by the high availability of easily accessible carbon sources (such as glucose and maltose) and by the absence of lignocellulosic substrates (Nurika 2020). Under these conditions, the detected CMC-depolymerizing activity may indicate, in addition to the non-enzymatic pathway, a basal constitutive expression of endoglucanases. Taken together, these findings highlight an adaptive strategy of *S. lacrymans.* The fungus maintains a minimal level of cellulolytic activity even when growing on simple sugars, ensuring readiness to exploit more complex carbon sources when they become available. In the presence of CMC, however, the cellulolytic system is fully activated, reflecting an ecological adaptation that underpins its efficiency in wood decay.

This aligns with previously reported substrate-induced expression profiles in basidiomycetes (Martinez 2009). Similarly, *S. lacrymans* increases production of phenolic metabolites and iron-reducing agents in response to cellulose availability, enabling effective depolymerization even in the absence of lignin. It has been shown that transcriptional and post-transcriptional regulation of enzyme systems in *S. lacrymans* suggests a nuanced control of cellulolytic expression (Nurika 2020).

### Contribution of enzymatic and non-enzymatic mechanisms in CMC degradation

To verify endoglucanase activity, we performed experiments using heat-inactivated culture fluid obtained from cultivation under conditions designed to suppress iron reductase expression—specifically, in the presence of simple sugars and the absence of complex lignocellulosic substrates. The observation that culture fluid loses its ability to reduce CMC viscosity after thermal denaturation (100 °C) suggests that the activity was diminished through the inactivation of endoglucanases, since iron reductase expression is not expected under such conditions. Since proteins typically lose function under these conditions, residual activity indicates the presence of oxidative mediators. This behavior also conforms to the CMF mechanism (Andlar 2018), in which extracellular chelators of Fe(III), such as oxalic acid, promote iron reduction and hydroxyl radical formation in the absence of enzymatic catalysis. This process is well documented for brown rot fungi, and *S. lacrymans* is known for high CMF activity due to active production of hydrogen peroxide and oxalic acid (Ritschkoff 1990). However, direct quantification of these compounds—e.g., H_2_O_2_ levels or Fe(III)-complexes—was not performed in this study. Future work should include spectrophotometric or chemical verification of CMF activity.

Although the dynamic viscosity changes between heat-treated and untreated culture fluids were less pronounced, they remained statistically significant. This reinforces the biphasic nature of substrate degradation in *S. lacrymans* S35 and highlights the biochemical resilience of its non-enzymatic oxidizers under stress conditions, such as elevated temperature.

Collectively, these insights support a hybrid degradation model in *Serpula spp.*, where efficacy depends on both enzyme induction and chemical stability of oxidative components. Such an integrative approach offers a foundation for standardizing cellulolytic activity assessment in brown rot fungi and may inform strain comparisons, antifungal development, and bioconversion optimization.

### Limitations

This study was conducted using a single strain of *S. lacrymans* under controlled laboratory conditions. Further studies involving purified enzymes and comparative analysis across strains are needed to validate the observed degradation mechanisms.

## Conclusion

This study provides several advances in the understanding of *S. lacrymans,* particularly in elucidating its physiological adaptations and biochemical mechanisms of cellulose degradation. First, we established that cultivation in a Murashige–Skoog salt-based medium supplemented with sucrose markedly improves biomass yield compared to traditional malt- or CMC-based formulations. This finding reflects strategies of competition and ecological niche adaptation, as successful antagonists must be able to compete under the conditions in which *S. lacrymans* is most active. Our results therefore provide a basis for modeling environments in which biological agents may effectively suppress the fungus. Moreover, studying the enzymatic activity of *S. lacrymans* is most reliable when the fungus is cultivated under optimized nutritional conditions, since sufficient biomass ensures reproducibility of assays, balanced mineral supply prevents distortions caused by nutrient limitations, and enhanced enzyme secretion allows the true spectrum and activity of cellulolytic systems to be revealed. Thus, optimized growth conditions are essential not only for physiological investigations but also for accurate evaluation of potential biological control strategies.

Second, we detected indications of basal endoglucanase activity in culture filtrates of *S. lacrymans*. Earlier studies generally described the enzymatic cellulolytic activity of this fungus as weak and strictly substrate-inducible, despite genomic evidence for GH12-type endoglucanase genes. Our observations suggest that *S. lacrymans* may sustain a minimal but persistent level of endoglucanase activity, potentially serving as a priming mechanism for subsequent oxidative degradation. While this interpretation requires further validation, it extends current knowledge and raises the possibility that hydrolytic enzymes in brown-rot fungi may exhibit low-level constitutive synthesis.

Moreover, our results revealed that *S. lacrymans* employs both hydrolytic enzymes and Fenton-like oxidative processes in cellulose degradation. This dual mechanism highlights the complexity of its decay system and aligns with strategies of mycoparasitism and production of inhibitory metabolites. Antagonistic fungi can potentially interfere with these pathways through secretion of chitinases, glucanases, and antibiotics. For the development of effective biological control, it is therefore essential to recognize that *S. lacrymans* relies on both enzymatic and oxidative mechanisms, each of which must be targeted.

Taken together, these findings advance our understanding of the biochemical and physiological traits of *S. lacrymans* and establish a foundation for the development of biological control strategies. Such approaches are urgently needed, given the limited efficacy of conventional chemical treatments, which typically act only on the wood surface and fail to eradicate the fungus within deeper layers of timber.

This study is limited to the analyzed *S. lacrymans* strain and the specific culture conditions applied. The growth and enzymatic activity measurements presented here are intended solely for comparative purposes and should not be interpreted as absolute or mechanistic indicators. Furthermore, the findings are not generalized to other strains or to potential industrial applications, but rather reflect the characteristics observed under the defined experimental framework.

## Data Availability

All data generated or analyzed during this study are included in this published article and its supplementary materials. Methodological details are available from the corresponding author upon reasonable request.

## Abbreviations

BSA: Bovine serum albumin
CMC: Carboxymethyl cellulose
CMF: Fenton mechanism
MS: Murashige–Skoog medium
*S. lacrymans*: *Serpula lacrymans*
